# A longitudinal study of the arterio-venous fistula maturation of a single patient over 15 weeks

**DOI:** 10.1007/s10237-022-01586-1

**Published:** 2022-05-25

**Authors:** Eamonn Colley, John Carroll, Simmons Anne, Thomas Shannon, Varcoe Ramon, Barber Tracie

**Affiliations:** 1 School of Mechanical and Manufacturing Engineering, The University of New South Wales, Sydney, NSW 2052 Australia; 2 Department of Surgery, Prince of Wales Hospital, Randwick, NSW 2031 Australia

**Keywords:** Dialysis, Vascular access, Arterio-venous fistula, Computational fluid dynamics

## Abstract

Arterio-venous fistula creation is the preferred vascular access for haemodialysis, but has a large failure rate in the maturation period. Previous research, considering the remodelling mechanisms for failure-to-mature patients, has been limited by obtaining the patient-specific boundary conditions at only a few points in the patient history. Here, a non-invasive imaging system was used to reconstruct the three-dimensional vasculature, and computational fluid dynamics was used to analyse the haemodynamics for one patient over 15 weeks. The analysis suggested evidence of a control mechanism, which adjusts the lumen diameter to keep the wall shear stress near constant in the proximal regions of the vein and artery. Additionally, the vein and artery were shown to remodel at different growth rates, and the blood flow rate also saw the largest increase within the first week. Wall shear stress at time of creation may be a useful indicator for successful AVF maturation.

## Introduction

There are over 2 million end-stage kidney disease (ESKD) patients who require kidney replacement therapy worldwide, and this is estimated to rise to over 5 million by 2030 (Liyanage et al. 2015). Haemodialysis, used by the majority (60–70%) of ESKD patients, requires vascular access, where blood can be taken out of the body (via cannulation) and pumped through an external dialysis machine, which filters the blood of waste and excess fluid before being returned. Vascular access is typically created via an arterio-venous fistula (AVF).

The AVF has among the highest failure rates of any elective surgical procedure that patients undergo, with recent clinical studies showing 25–60 failure usually occurs due to insufficient dilation of the vessel and/or stenosis, which results in inadequate blood flow rates capable of haemodialysis. High flow rates are associated with successful AVF maturation, and there is also an increase to cardiac output and total redistribution of blood flow; in a typical successful maturation, the arterial flow increases approximately tenfold (25–270 mL/min) within the first day and further increases to approximately 570 mL/min over the next 4–8 weeks (Dixon 2006).

For a typical successful maturation, there is a large increase in flow in the first few days post-creation (Dixon 2006) which is mostly attributed to the large dilation in the vein (approximately 60% increase in diameter), but also to the arterial dilation (approximately 20% increase in diameter).

A sustained increase in flow will lead to an increase in vessel diameter, regulated by the endothelial cells which line the vessel walls. If the endothelial cells sense an increase in wall shear stress (WSS), the vessel remodels outward to lower the WSS back to a baseline level (Girerd et al. 1996), yet different baseline levels for WSS exist in various parts of the human body and between different species of animals (Cheng et al. 2007). The direction of WSS corresponds to the local, near-wall flow direction,

1 $$\begin{aligned} {\text{WSS}}=\mu \cdot \dot{\gamma } \end{aligned}$$

where $\mu$ is the fluid viscosity and $\gamma$ is the wall shear rate. Wall shear rate is defined as the difference between adjacent mesh-point velocities, divided by the distance between them.

Studies to monitor newly created AVFs have reported elevated WSS in the artery when compared to baseline (pre-creation) values (Ene-Iordache et al. 2003). It would follow that the artery will increase in lumen diameter to lower the WSS back to baseline, which has been a hypothesis of previous AVF studies (Javid Mahmoudzadeh Akherat et al. 2017). It is generally accepted that arterial vasculature seeks to maintain constant WSS at a preferred, homeostatic value (Humphrey 2008), with the presence of a control mechanism demonstrated by Le Noble et al. (2005). This control mechanism is dependent on a ‘set point’ in which any deviation will result in remodelling of the artery; an increase in WSS from the set point will result in outward remodelling to reduce the WSS back to the set point (Langille 1996). The set point varies in different parts of the arterial tree due to pulsatility in flow waveforms from reflections, and this naturally increases or decreases the mean WSS. However, Ene-Iordache et al. (2003) has shown that the mean WSS remains elevated from the baseline in the radial artery for the entire maturation period, despite the diameter increasing.

Patient-specific AVF longitudinal studies are limited. In 2013, Sigovan et al. (2013) published a longitudinal AVF study, using computational fluid dynamics (CFD), on humans using non-contrast MRI^1^ techniques and quantified the 3D geometric and haemodynamic changes. Significant geometrical and haemodynamic changes between the first scan at five days and the second scan at one month (for three patients) were noted, but a clear relationship between WSS and vascular remodelling was not identified. In a similar study, He et al. (2013) could not distinguish a defined relationship between disturbed flow and lumen changes. Bozzetto et al. (2018) scanned one AVF at 1 and 6 weeks post-creation using contrast-free MRI. They showed a general outward remodelling in the vein and proximal artery, while the distal artery remained the same.

Temporal data are needed to determine the relationship between haemodynamics and vascular remodelling, yet establishing a trend with minimal data points is challenging. A limitation of previous longitudinal studies is obtaining patient-specific data across a number of time points. Using our previously described system (Colley et al. 2018), we here outline a study in which one patient was scanned weekly, for 15 weeks. The relationship between WSS and outward remodelling was investigated, in addition to the WSS metrics, and due to the fifteen-weekly scans, we were able to further explore the vascular remodelling stability for this patient.

## Methods

### Scanning system

A scanning and processing procedure was developed by our team specifically for geometric tracking and CFD modelling of AVFs, using a freehand ultrasound set-up combining B-mode scanning with 3D probe motion tracking as previously described (Carroll et al. 2020; Colley et al. 2018). A 3D tracking camera is mounted on the ultrasound, in line of sight with the patient as the scanner moves the tracked probe over the scan target location, sweeping along the vasculature to create a high-density stack of B-mode frames containing the lumen geometry. This stack is converted into a continuous volume as a 3D voxel grid, filling gaps between frames, with the vasculature geometry isolated through segmentation. Geometric calibration was verified through scanning a cross-wire phantom; comparing measurements of the scan with the actual measured values showed a mean error of 2.5%. Transient flow waveforms are recorded at the boundaries, synchronised with electrocardiography (ECG) and automatically digitised, forming realistic boundary conditions for the CFD models.

Volume flow rate waveforms were measured at the proximal artery (*PA*) and distal artery (*DA*) boundaries of the geometric scan sweep, using transient centreline peak velocity detected with spectral Doppler ultrasound. These measurements using the Mindray L14-6NS probe are taken as far proximal and distal from the anastomosis as feasible for each patient case. As measurements at each location were taken sequentially, temporal re-synchronisation was required. Three-lead ECG was overlaid in real time while recording the Doppler spectra at each boundary location. Multiple periods of the peak velocity waveforms were captured, using the R peak location of the QRS complex from the ECG waveform (corresponding to ventricular contraction), to globally define endpoints for each period. Transient flow rates *Q*(*t*) were determined from centreline velocity $V_{p}(t)$ and measurements of cross-sectional diameters *D*, using the relationship $Q(t)=u_{t} \cdot \frac{\pi D^{2}}{8}$, where $u_{t}$ is the peak velocity as a function of time of the cardiac cycle and *D* is the diameter which is measured from the ultrasound B-mode image; this assumption is an accepted limitation of the model.

### Computational modelling

Patient-specific boundary conditions are obtained to numerically solve the pressure and velocity at discretised cells inside the geometrical domain. The governing incompressible Navier–Stokes equations were solved using finite volume code in FLUENT 16.2 (ANSYS Inc.), where the SIMPLE algorithm is used to solve for the pressure–velocity coupling. A second-order upwind scheme spatially discretised the momentum and pressure variables, and temporal discretisation was achieved using a second-order implicit scheme. Throughout this work, blood is assumed to be an incompressible fluid with a constant density of 1060 kg/m$^3$ and treated as a non-Newtonian fluid, and the Carreau model is employed to describe the viscosity behaviour:

2 $$\begin{aligned} \mu =\mu _{\infty C}+\left( \mu _{0}-\mu _{\infty C}\right) \left[ 1+(\lambda \dot{\gamma })^{2}\right] ^{\left( n_{c}-1\right) / 2} \end{aligned}$$

where Cho and Kensey (1991) $\dot{\gamma }$ is the shear rate, $\lambda$ is a time constant = 3.313, $\mu _{0}$ is the viscosity at zero shear rate = 0.056 $\mathrm {Pa} \cdot \mathrm {s}$ and $\mu _{\infty C}$ is the viscosity at infinite shear rate = 0.00345 $\mathrm {\,Pa} \cdot \mathrm {s}$.

The turbulence is computed using the k-$\omega$ shear stress transport (SST) model; peak Reynolds numbers (estimated for each geometry) are in the range of 500–2200, yet it has been previously reported that there is transitional to turbulent flow present in the venous swing segment (the region just past the anastomosis) of the AVF (Bozzetto et al. 2016; Browne et al. 2015; Ene-Iordache et al. 2015a). This model has been shown to have good accuracy for transitional flows and flows near the wall in carotid artery bifurcations (Tan et al. 2008), aneurysms (Tan et al. 2009) and blood damage analysis (Goubergrits et al. 2016), which have similar wall shear stress analyses as AVFs. The vessel walls are set as rigid with a no-slip condition. A rigid-wall assumption is acceptable for identifying key features in an AVF, but with the expectation that the values of wall shear stress may be over-estimated by up to 10% in the proximal regions (McGah et al. 2014).

Each patient AVF case has a time-varying velocity profile that is set at the proximal inflow artery and outflow artery, which are measured via pulsed-wave Doppler at the specified locations each week. The proximal outflow vein is set with a 0-Pa pressure outlet. The simulation is calculated for three cardiac cycles with a time step of 0.001s, which was verified with a time-step independence study on two cases, resulting in a maximum WSS error of 0.3%. Each simulation was computed on a cluster, running on 64 CPUs at a speed of 2.2 GHz each. On the last cycle, the full transient solution was saved every ten time steps for flow analysis, as in previous similar work (Fulker et al. 2018).

**Fig. 1:**
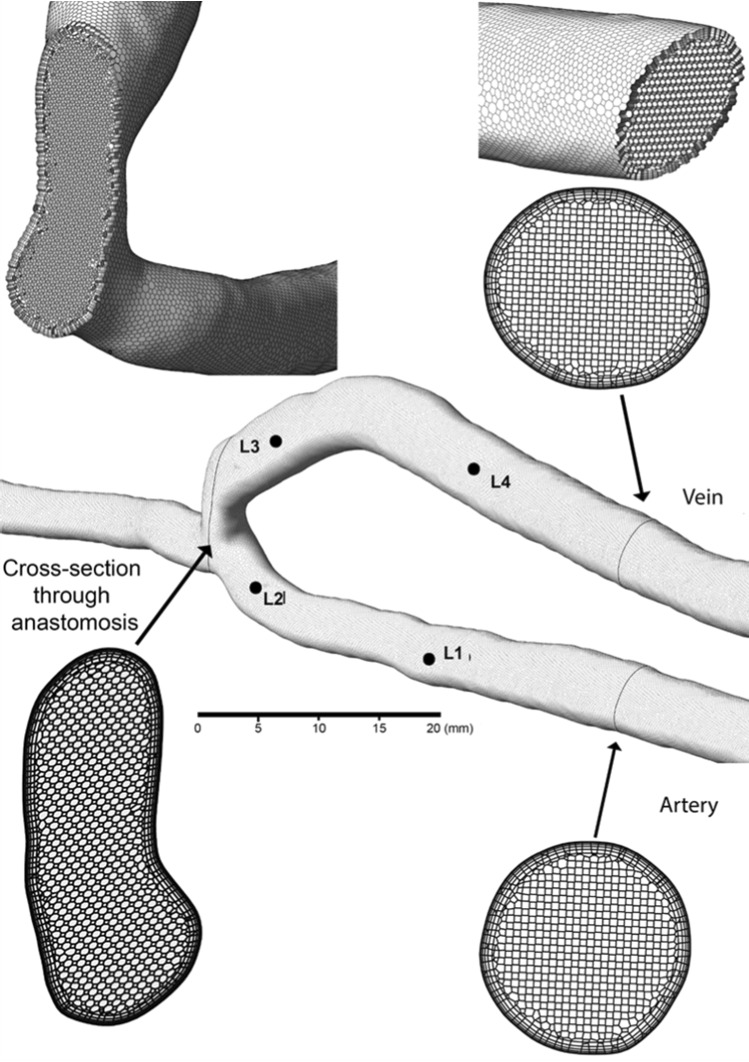
Unstructured mesh. The mesh is generated with tetrahedral elements and then converted to polyhedral elements. The cross section of the polyhedral mesh at the vein, artery and anastomosis displays the mesh resolution at the core and boundary layers; element height starts at 0.025 mm near the wall. TAWSS values at locations 1–4 are used to test for grid independence

A surface model for each scan is produced and the mesh created using ICEM (ANSYS Inc); each unstructured tetrahedral mesh is generated with five high-density prism boundary layers placed at the wall to resolve the near-wall velocity gradients such that $y^{+}<1$ to ensure wall modelling accuracy and then converted to polyhedral elements. An example of the mesh is shown in Fig. 1.

To verify grid independence, the grid convergence index (GCI) (Roache 1993) is calculated. The flow is computed for three meshes, with a grid ratio of more than 1.1 so that discretisation error can be differentiated from other error sources. Similar node spacings and grid densities were generated in all geometries, and the volume cell size was reduced systematically to produce three different meshes for each geometry. Note that the total number of cells differs between the patients due to the length of segment captured; however, the total mesh size ranged from 600K elements to 1.2M elements. The grid refinement ratio (r) is defined for unstructured grids as $r_{ij} = (N_j/N_i)^{(1/D)}$, where D is the dimension of the flow domain, which in this case is three. The discretisation error is estimated for time-averaged wall shear stress ($\phi$ = TAWSS), in four different geometrical locations (L1, L2, L3, and L4) for two different patient-specific geometries, where TAWSS is the average of the wall shear stress over the cycle. Discretisation errors in the calculated TAWSS at *L* = 1:4 are 1.494%, 0.228%, 2.743% and 0.182%, respectively, for the fine-grid solution. Therefore, a medium grid was chosen. For each scan, the centrelines through the vasculature are generated and the anastomosis is defined as the intersection of these centrelines, and this point is used as a global reference for each scan and assumed to not change during the maturation period.

### Wall shear stress metrics

Ku et al. (1985) provided evidence that atherosclerosis lesions occur where the flow near to the wall is oscillatory in behaviour; oscillatory shear index (OSI) was formed to describe this flow behaviour. OSI was later modified (He and Ku 1996) for use in three-dimensional flows, as shown by Eq. 3:

3 $$\begin{aligned} {\text {OSI}} = \frac{1}{2}\left( 1 - \frac{|\int _{0}^{T} \vec{\tau _{w}}\mathrm{d}t |}{\int _{0}^{T} |\vec{\tau _{w}} |\mathrm{d}t}\right) \end{aligned}$$

where $\vec{\tau _{w}}$ represents the instantaneous WSS vector, t is the time and T is the duration of the cardiac cycle. OSI represents the cyclic departure of WSS from its predominant axial direction; it is a dimensionless change in WSS direction ranging from 0 to 0.5, with the denominator being the time-averaged wall shear stress (TAWSS). As a time-averaged RANS model is used here, only the variation of the mean flow is taken into account rather than any small-scale turbulent fluctuations. Multiple theories exist that correlate a (WSS) disturbance metric to the aetiology of neointimal hyperplasia (NIH). The low/oscillatory WSS theory has been suggested to correlate with future sites of stenosis due to NIH (Ene-Iordache et al. 2015b), whereas some authors have suggested that high WSS (Carroll et al. 2011) is correlated with development of disease.

Further research by Peiffer et al. (2013) investigated multi-directional flow, with the hypothesis that WSS components acting transversely to the mean vector are pro-atherogenic. They devised a new metric (transWSS), which is described by Eq. 1.6, and showed lesion prevalence correlated strongly with transWSS and no correlation supported the low/oscillatory WSS theory. However, it should be noted that transWSS does not completely characterise WSS behaviour, and OSI is still needed to distinguish purely forward flow and pulsatile flow with reversal.

4 $$\begin{aligned} { transWSS = \frac{1}{T}\int _{0}^{T} \left|\vec{\tau _{w}} \cdot \left( \mathbf {n} \times \frac{\int _{0}^{T} \vec{\tau _{w}}\mathrm{d}t }{\left|\int _{0}^{T} \vec{\tau _{w}} \mathrm{d}t\right|} \right) \right|\mathrm{d}t }. \end{aligned}$$

### Patient information

Patients were recruited at Prince of Wales Hospital, Sydney, Australia, with approval from the Human Research Ethics Committee (HREC ref: 15/063), as part of an going study (Colley et al. 2020). A 56-year-old male patient, scheduled for fistula creation surgery (and not currently on dialysis), agreed to attend the clinic on a weekly basis to enable scans to be conducted. The patient underwent a pre-scan before surgery, and subsequently, a radio-cephalic fistula was formed surgically where the radial artery and cephalic vein were dissected off their bed and anastomosed to each other in an end-to-side configuration. Pre-operatively, the cephalic vein had an average diameter size of 1 mm and more than 2 mm when a proximal venous tourniquet was applied.

**Fig. 2:**
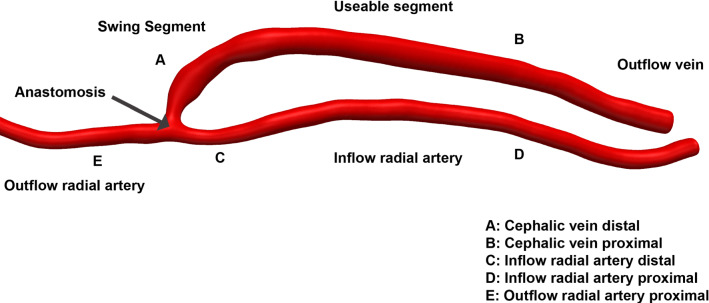
Anatomical regions of the three-dimensional vasculature. A volume of the AVF lumen is created from the 3D freehand ultrasound system, and the focus regions are defined

The 3D freehand ultrasound system (Colley et al. 2018) was used to obtain the lumen geometry as shown in Fig. 2. The AVF is defined for five different regions (A–E): swing segment of the distal vein, the proximal vein, inflow artery distal, inflow artery proximal and the outflow artery proximal.

**Fig. 3:**
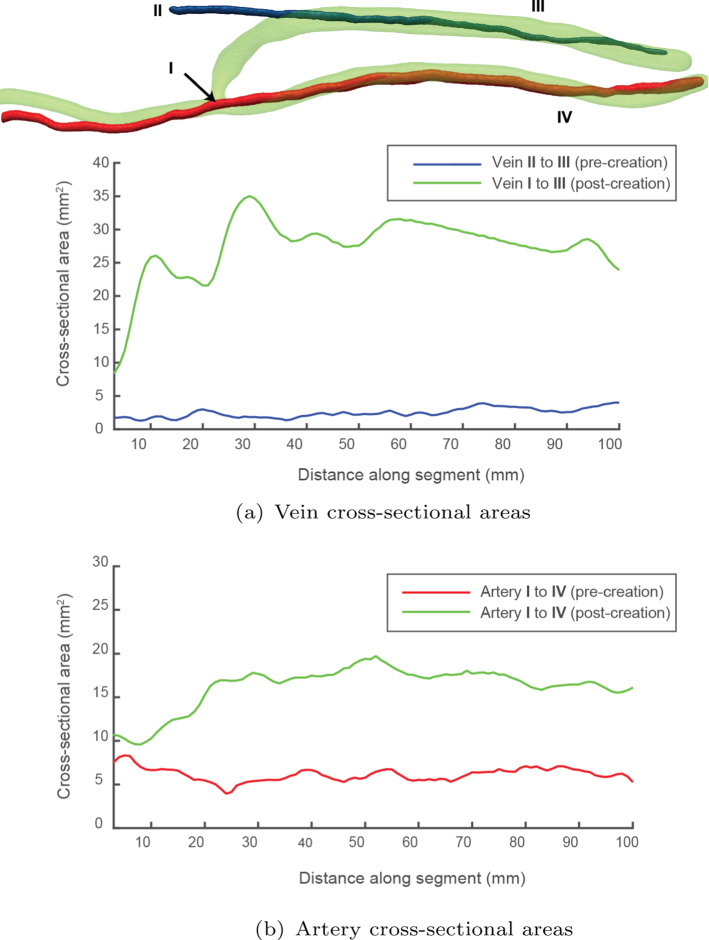
Comparison of the vasculature at baseline and at 1 week post-surgery. The cephalic vein (blue) is surgically attached to the radial artery (red) to create an AVF (green). A comparison at baseline to 1 week post-surgery shows the large difference in cross-sectional areas by flow-induced remodelling. For the baseline artery and vein, the cross-sectional areas are taken from the approximate location where the AVF was created

## Results

### Geometric changes

The vein and artery of the patient were scanned before surgery and then at seven days post-surgery. A reconstruction of the geometry and the cross-sectional area comparison is shown in Fig. 3. There is an immediate increase in cross-sectional area of both vein and artery. The cross-sectional area of the vein has increased by 1050% (from 2.7 to 31.05 mm$^2$) in the useable segment (for dialysis cannulation), while the juxta-anastomosis region (where the vein meets the artery) has tapering due to the fixed size from the sutures. The proximal artery increased 280% (from 5.1 to 17.08 mm$^2$) in the proximal location, but similar to the vein, the cross-sectional area is lower in the distal region.

**Fig. 4:**
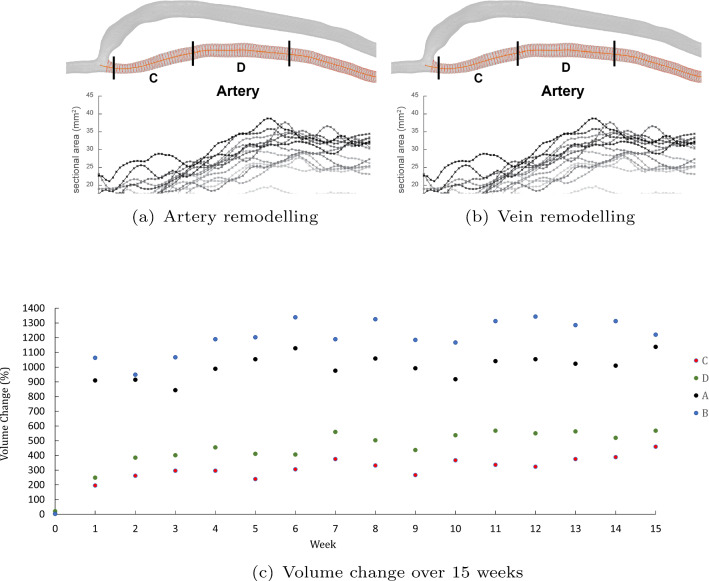
Longitudinal vascular remodelling. Longitudinal remodelling of the patient’s AVF over 15 weeks is shown for both artery and vein. Each point on the graph corresponds to the cross-sectional area, sampled at 1-mm distances along the centrelines of the vasculature. A greyscale is used to represent the weekly changes, with week 1 corresponding to a lighter shade and week 15 darker. The volume of each segment (**a–d**) is compared to the baseline level and fitted to a line of best fit to demonstrate the trend. **a, c** Anastomosis to 40 mm along the centreline. **b, d)** From 40 to 80 mm along the centreline from the anastomosis

A comparison of the geometric changes in both the vein and artery is shown in Fig. 4 over 15 weeks. The cross-sectional area is calculated at 1-mm intervals along the centreline of the vasculature. A greyscale is used to represent each of the weeks, where week 1 corresponds to lighter colours and week 15 is darker. There is an immediate increase in the artery cross-sectional area from week 1 to week 2 in the proximal region, which then steadily increases as the weeks progress.

A volume of each of the segments (A to D) is calculated for each week. The volume is calculated by integrating the cross-sectional areas along the centreline. Lines of best fit are provided to demonstrate the trend across weeks 1–15.

While the artery continues to remodel over time, the vein has little change in comparison: 200% between the first and last weeks in the proximal artery segment (D) compared with 20% in the proximal vein segment (B).

### Blood flow rates

**Fig. 5:**
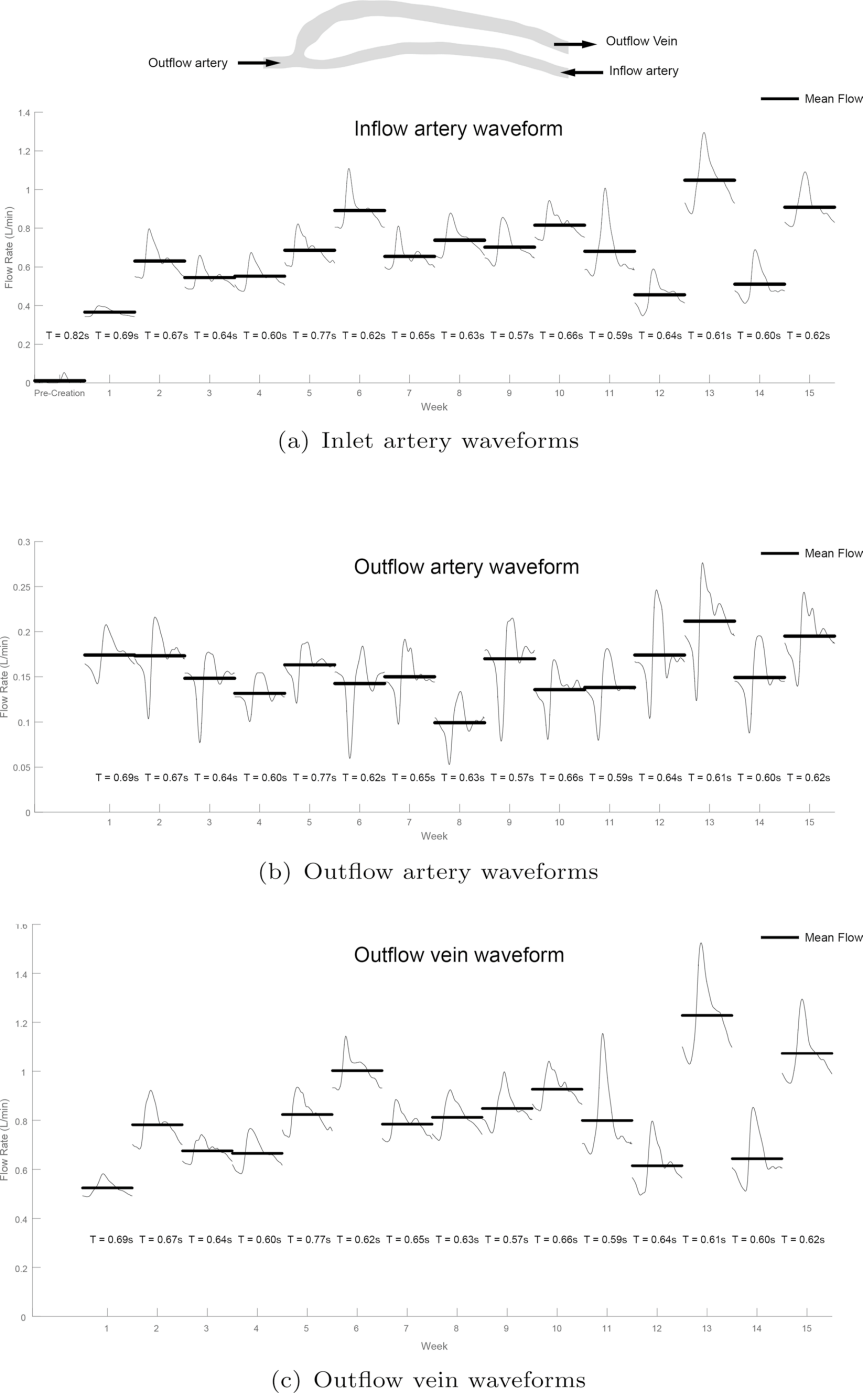
Transient waveforms. The transient waveforms are measured with Doppler ultrasound and are shown for the inflow artery, outflow artery and outflow vein. At pre-creation, the flow rate in the inflow artery is pulsatile with a mean flow rate of 10 mL/min. There is an apparent increase in the flow rate for the inflow artery and outflow vein, but the outflow artery stays approximately constant. It should be noted that after week 10, there are larger fluctuations in the flow rates. Each flow rate is normalised with the cardiac cycle, where T represents the cycle length in seconds (s)

Transient flow rates were obtained via Doppler ultrasound at the inflow artery, outflow artery and outflow vein, and the measured waveforms at each week are shown in Fig. 5. As the outflow artery has retrograde flow, the outflow vein flow is a composition of the inflow artery and outflow artery. The outflow artery still consists of the initial peak velocity as it travels through the ulnar artery and around the palmar arch of the hand, but is then rapidly decelerated when the flow collides with the inflow artery flow near to the anastomosis.

Pre-creation, the inflow artery waveform was highly pulsatile, as is expected in distal vasculature. Once the AVF is made, the waveform has one major flow peak (from the inflow artery) and then a smaller second reflection peak from the outflow artery. The mean flow rate at the inflow artery, represented by black bars, is 10 mL/min at pre-creation and increases to a mean flow rate of 360 mL/min at week 1. At pre-creation, there were no other branches in the radial artery and hence the inflow is equal to the outflow.

As the maturation progresses, there is an increase to the flow rate in the inflow artery. The outflow artery stays approximately constant, and hence, the outflow vein increases at approximately the same flow rate as the inflow artery. After week 10, there are significant fluctuations (of the order of 500 mL/min) in the inflow artery flow rate.

**Fig. 6:**
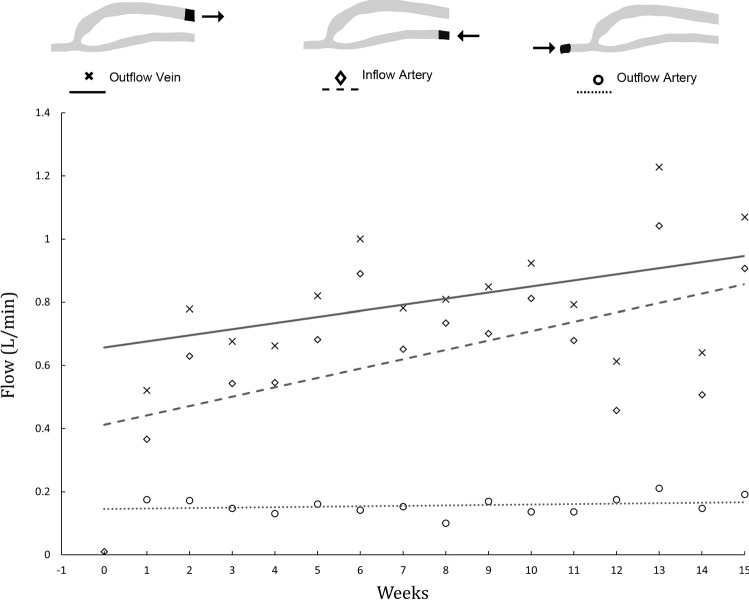
Mean flow rates. The mean flow rate is shown for the inflow artery, outflow artery and outflow vein and lines of best fit added to demonstrate the trend. There is a 36-fold increase from pre-creation to week 1 in the inflow artery (10 mL/min to 360 mL/min). The outflow artery has retrograde flow which remains approximately constant, and accounts for approximately 20% of the flow in the vein

The mean flow rates of the three boundaries at each week are shown in Fig. 6. While the mean flow rate of the artery inflow and vein outflow increases over the duration of the maturation, the artery outflow stays approximately constant. The outflow artery accounts for approximately 20% of the flow in the vein at the beginning of the maturation, but this proportion decreases as time progresses.

The outflow vein has a mean flow rate of 520 mL/min at week 1, with a diameter of over 6 mm. By week 2, the flow rate has increased to 780 mL/min which is within the requirements for dialysis purposes. From the fitted curve, the average flow in the vein continues to increase over the weeks, approximating 900 mL/min at week 15.

**Fig. 7:**
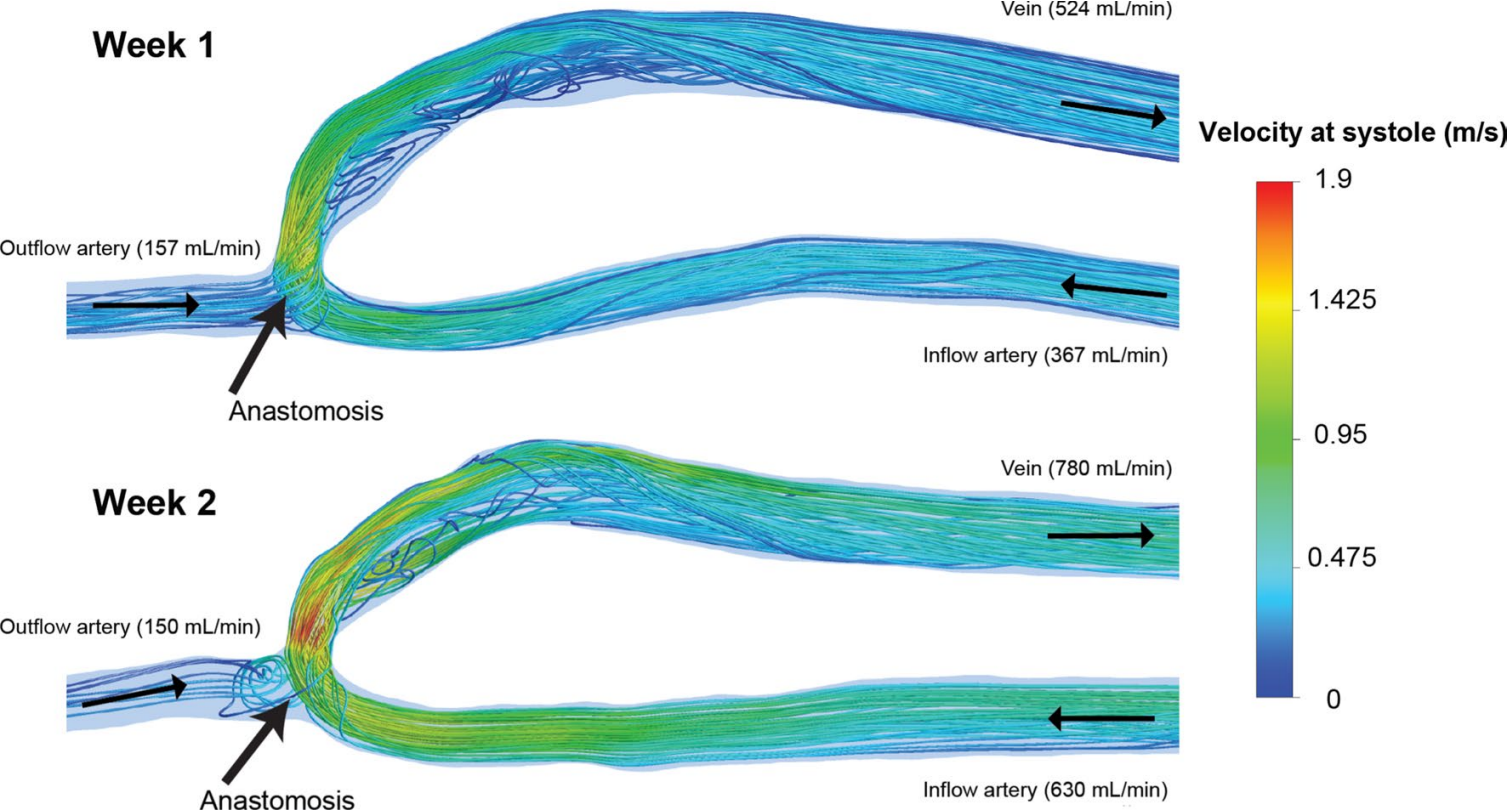
Velocity streamlines at systole for week 1 and week 2. The velocity streamlines are shown for week 1 and week 2, where the majority of the remodelling happens in the maturation. There is a large increase in flow between the 2 weeks. Note that the flow in the outflow artery is retrograde

There were large changes in mean blood flow rate between week 1 (367 mL/min at the inflow artery) and week 2 (630 mL/min at the inflow artery), and hence, the velocity streamlines are shown for these two time points in Fig. 7. At week 2, the inflow artery blood flow rate accounts for a larger proportion of the venous flow rate, than at week 1. This is due to the outflow artery having approximately the same flow rate between the weeks (157 mL/min at week 1 and 150 mL/min at week 2).

At week 1, there is a high-velocity jet which is skewed to the outer wall of the venous swing segment, whereas there appears to be flow re-circulation and separation near to the inner wall, which does not reattach until further downstream in the vein. The Reynolds number at the inflow artery has an approximate peak value of 730 indicating laminar flow, but the peak Reynolds number at the venous swing segment is much higher (approximately 1700). At week 2, the inflow artery has a peak Reynolds number of 1000, but within the swing segment the peak Reynolds number is approximately 2250. This indicates the flow is within the transitional regime, yet, the pulsatile nature of the blood flow is known to have an influence on the regime threshold and transitional flow may occur at a lower Reynolds number.

The flow at week two appears to be more disturbed through the anastomosis and venous swing segment, with much higher-velocity jets due to the increased flow, but very little diameter change when compared with week 1.There is also a larger re-circulation zone present in the floor of the anastomosis, and throughout the swing segment.

Sites of disturbed flow have been observed to coincide with disease development in which the magnitude and multi-directionality of the WSS can disrupt endothelial function. To quantify the disturbance seen in the maturation, WSS metrics are computed in the following section.

### Wall shear stress metrics

**Fig. 8:**
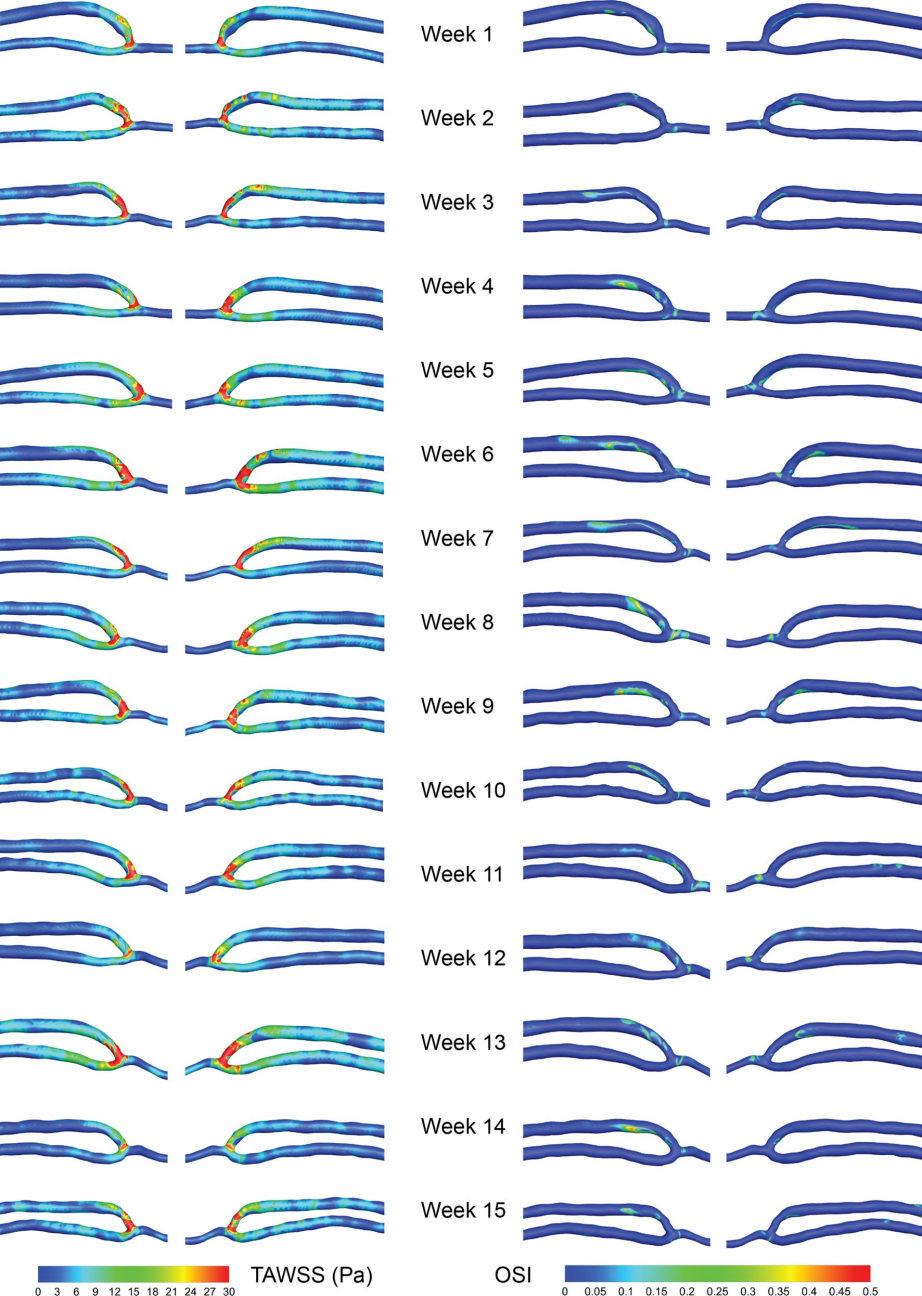
TAWSS and OSI contours—weekly changes. Time-averaged wall shear stress (TAWSS) is shown on the left and the oscillatory shear index (OSI) on the right, displaying both the medial and lateral view. High TAWSS is seen in the vein swing segment and anastomosis region. High OSI values are seen on one side of the AVF, typically in the vein swing segment and just after. Note that the geometry is not to scale

The 3D contours of both time-averaged wall shear stress (TAWSS) and the oscillatory shear index (OSI) are shown for each week in Fig. 8. High TAWSS is found in the vein swing segment each week and lesser values in the distal inflow artery. These high values are due to high shear rates as the cross-sectional area decreases. In the vein swing segment, values of TAWSS are unstable week to week, as the velocity jet through the anastomosis is skewed to the outer wall, but varies in location due to the different magnitudes of incoming flow each week. Relatively lower TAWSS is found in the proximal artery and vein regions.

High values of OSI occur in the swing segment, and just after as the vein straightens out, with the majority found on one side of the vessel due to the out-of-plane curvature of the vein and artery. The contour patterns vary a small amount, but remain in approximately the same locations week to week.

**Fig. 9:**
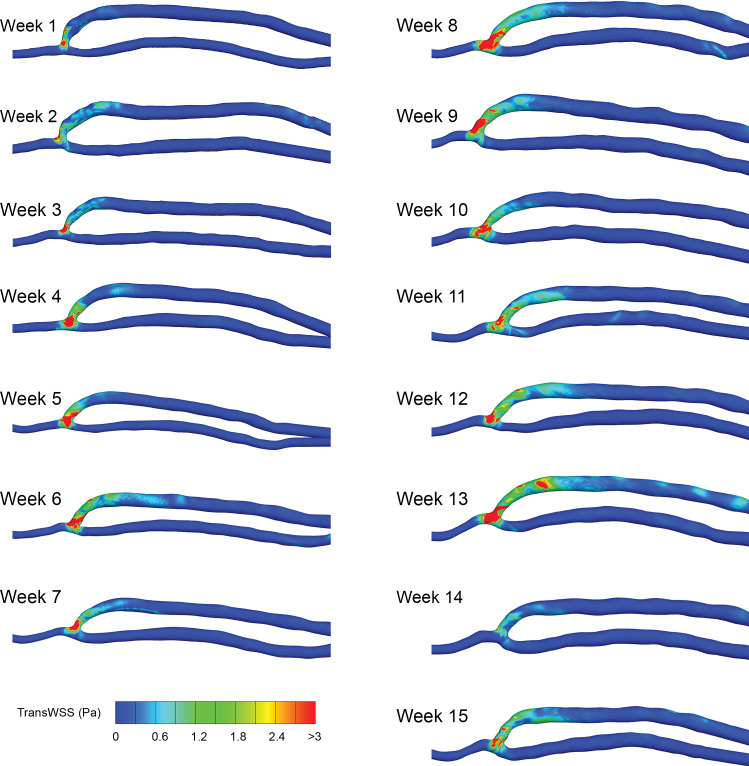
transWSS contours—weekly changes. The transWSS is shown for each of the weeks. Note that the geometry is not to scale

The multi-directional flow near the wall was quantified using the transWSS metric, and the contours are shown in Fig. 9. The highest values (>3 Pa) are in the anastomosis region, due to the collision of the inflow and outflow artery flows. There are also places of mid-range (0.6–1.2 Pa) and high transWSS in the venous swing segment, but these reduce downstream. The regions of high multi-directional WSS are in similar locations week to week and do not appear to change in area size significantly.

### Vascular remodelling

**Fig. 10:**
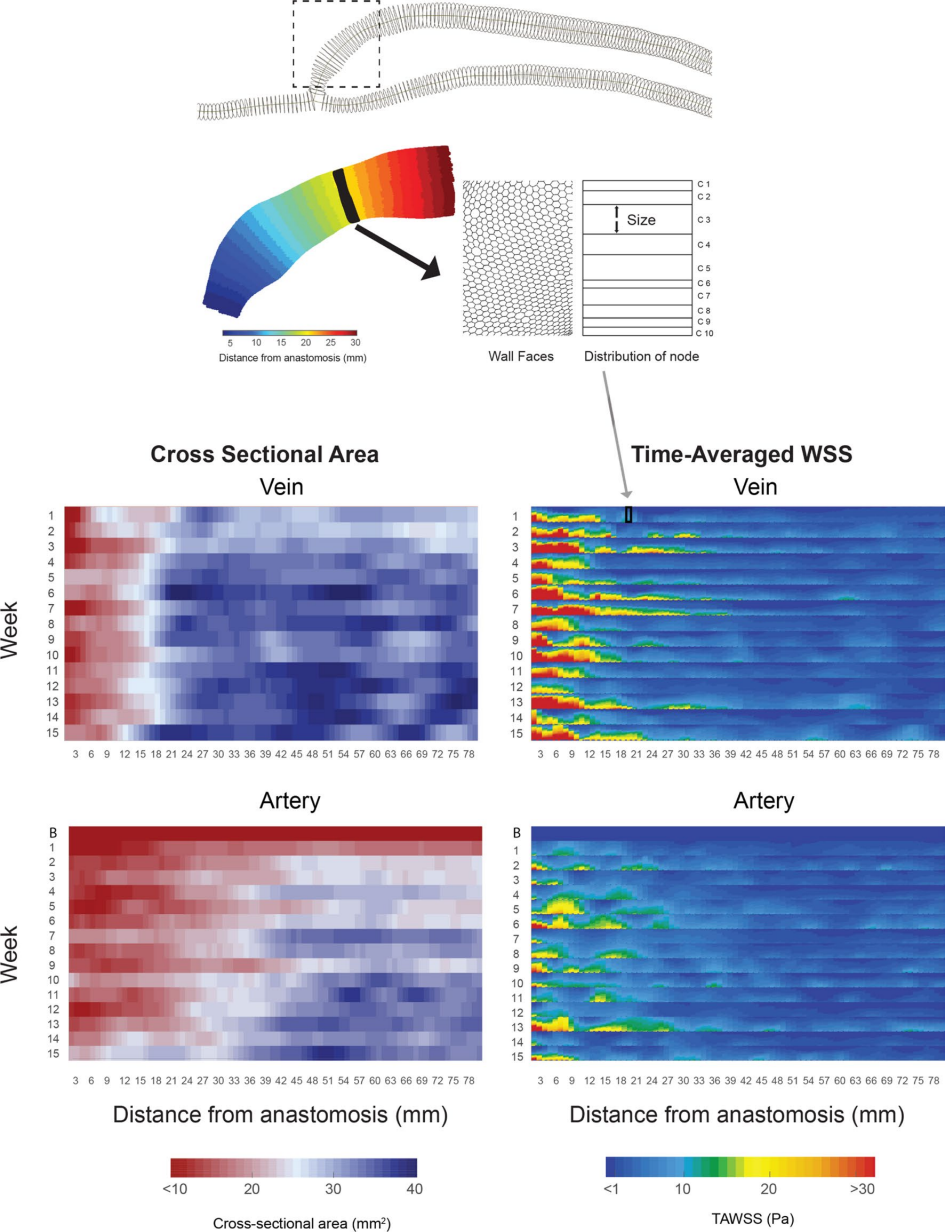
Geometric and TAWSS comparison. Top: The vasculature is divided into sub-volumes (nodes), spaced 1 mm apart and an example node is shown in the highlighted region in the figure. For each node, the nearest face cells on the wall, which make up the surface area of the sub-volume, are represented by a 2D rectangular box, shown here in black. The wall faces within this region shown in black, which contain the TAWSS values, are sorted via a histogram function, where the size indicates the bin count and the colour by the magnitude of the TAWSS in that bin. This allows a comparison of WSS distribution along with the area distribution, over time. Bottom: The cross-sectional area (left) at each node is shown for weeks 1–15 in the vein and from baseline to week 15 in the artery. TAWSS (right) shows large values near the anastomosis in both the vein and artery, with much lower values after 40 mm

Further analysis is required so that the TAWSS can be quantified and a point-to-point comparison can be made at each week, as shown in Fig. 10.

There is a large increase in TAWSS from baseline (average 0.4 Pa) to week 1 (average 2.5 Pa) in the artery, and this value remains elevated in the subsequent weeks. In the proximal segment, further from the anastomosis, the TAWSS is lower, with still some variation within the nodes due to curvature of the vessel. There are localised regions of high TAWSS (more than 15 Pa), but the high values do not stay in the same location week to week and remain within 40 mm of the anastomosis.

Similar to the artery, the proximal vein segment shows variation of TAWSS within the nodes, but remaining elevated week to week. There are also much larger stresses on the wall in the swing segment, covering a large proportion of the surface area, especially less than 20 mm away from the anastomosis. These values settle by 40 mm along the centreline in most weeks.

There are significant increases in the cross-sectional area for the artery, particularly further than 40 mm away from the anastomosis. In the subsequent weeks, the proximal artery remodels from an average of 24–34 mm$^2$, but clearly shows localised, rather than uniform, remodelling. The outward remodelling progressively increases closer to the anastomosis for the duration of the 15 weeks.

The vein remodels outward in the first 3 weeks in the proximal region, but then little remodelling is seen for the duration of the maturation. Little to no remodelling is seen in the venous region between the anastomosis and 20 mm away, and then, there is a defined transition, where the cross-sectional area is relatively larger.

## Discussion

The results for this patient show that the majority of the geometric remodelling occurs for both the vein and artery within the first 2 weeks. Within the first week, the vein has increased by 1050% in the useable segment, and the artery increases by 280%, when compared with the baseline (pre-creation) values. The blood flow rate also has the largest increase within the first week, showing an increase from a mean blood flow rate of 10 mL/min to 360 mL/min in the inflow artery. The outflow vein has a mean flow rate of 520 mL/min at week 1 and has increased to 780 mL/min by week 2. By clinical definition of ‘The rule of sixes’ (the vein is more than 6 mm in diameter, less than 6 mm from the skin surface, and more than 600 mL/min of flow), this patient would be deemed a successful maturation by week two.

The flow showed much higher fluctuations week to week than the geometry. High flow disturbances were found in the venous swing segment of this patient at each week, caused by the inflow artery flow colliding with the retrograde flow in the outflow artery. As the anastomosis configuration tapers in the venous swing segment, there is evidence of a velocity jet phenomena present through this region, as well as recirculating flow. The variation in flow rates from week to week is unlikely to be entirely the result of the Doppler measurement error; this patient was concurrently being evaluated for a kidney transplant, in which the examinations occasionally took place prior to our scheduled scanning session. These examinations started in the second half of the longitudinal study and may contribute to the outliers seen in the data, particularly, for example, the data seen in week 13. Additionally, ESKD patients are often given medications for the management of kidney disease which would affect peripheral resistance and the blood flow waveform.

Little to no remodelling is seen in the venous region between the anastomosis and 20 mm into the vein, where there is a defined transition and the cross-sectional area is relatively larger. In appearance, the vein has heterogeneous variation of the area along the segment, which corresponded to high and disturbed flow. In the artery, there are lower cross-sectional area variations along the investigated segment, but current results do not demonstrate homogeneous remodelling, as reported by other studies (Sigovan et al. 2013). The vein and artery are seen to remodel outward at different rates. In the proximal segments, the artery continues to remodel over the fifteen weeks (200% change in volume), but the vein has little change in comparison (20% change in volume).

WSS results for this patient show that values are not restored to the pre-creation baseline set point as previously suggested (Javid Mahmoudzadeh Akherat et al. 2017), but fluctuate around a new, higher value. This demonstrates a possible control mechanism in which the diameter is adjusted to maintain WSS within a narrow range of the new set point. The results suggest that at time of AVF creation, the vasculature behaves similar to arteriogenesis, as similar shear rates patterns were observed in early stage arterial system development (Le Noble et al. 2005). Fluctuations of shear were also observed in a previous study (Le Noble et al. 2005) and had ‘constancy’ around a value.

Wall shear stress at time of creation could be a possible predictor for AVF success or failure. TAWSS values for the patient in this study do not change significantly from the measured value in week 1, despite large increases in flow and cross-sectional area. The TAWSS measured in the proximal inflow artery fluctuates around 3 Pa with a range of 1.5 Pa, which is elevated from the baseline level of approximately 0.4 Pa. These values agree with other WSS measurements in human radio-cephalic AVFs (Ene-Iordache et al. 2003), which also show elevated (from baseline) WSS during the maturation period.

To quantify the flow disturbance effect on WSS, OSI and transWSS were calculated at each week. The results show that high values of OSI and transWSS are confined to the anastomosis and swing segment region, which agrees with other patient-specific CFD studies (Ene-Iordache et al. 2015b). As the majority of these studies were taken at single-time points, there were not the data to support the theory that disease developed at these locations. It was found that in this patient continued exposure to flow disturbances, in the form of WSS metrics (OSI and transWSS), but this did not lead to severe NIH or disrupt the VA patency, at 15 weeks.

**Fig. 11:**
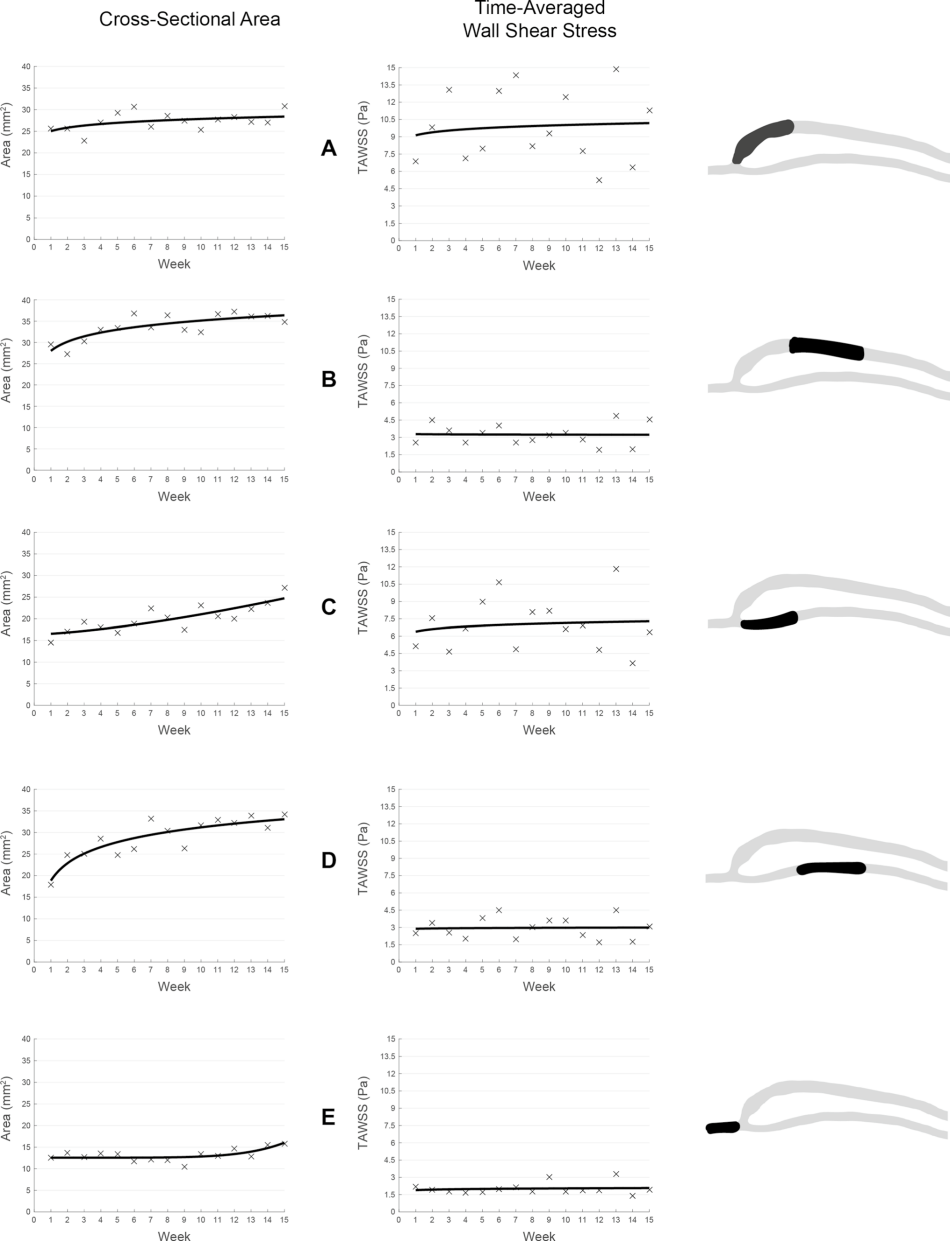
Vascular remodelling. Various segments of the AVF are shown and their associated changes in the spatially averaged cross-sectional area and time-averaged wall shear stress (TAWSS). Segments further from the anastomosis (**b, d**) have less weekly fluctuations in TAWSS and have constancy of approximately 3 Pa. Segments closer to the anastomosis (**a, c**) have a much higher baseline value and larger fluctuations. Trends are shown via lines of best fit through the data points

To explore the relationship between outward remodelling and TAWSS, the TAWSS is spatially averaged over the various segments (A to E) as shown in Fig. 11. Immediately, it is apparent that the AVF remodels at different rates in different regions, likely due to proximity to the anastomosis. Even though there are different rates of outward remodelling throughout the vein and artery, TAWSS either increases or decreases, but rather fluctuates around a value.

In the proximal outflow vein (segment B), there is an increasing trend in the cross-sectional area, but with a small rate of change. The spatially averaged TAWSS fluctuates around 3 Pa with a range of 1.5 Pa. The proximal inflow artery (segment D) has the same TAWSS trend (constancy of 3 Pa with a range of 1.5 Pa), but has a much larger rate of change in the cross-sectional area over the weeks.

There is a larger range of temporal fluctuation (week to week) of TAWSS in regions near to the anastomosis. In the distal region of the inflow artery (segment C), the TAWSS is much more elevated than the proximal region of the artery with an average difference of 3.5 Pa. Despite the large temporal fluctuations, they appear to still fluctuate around a value of 7.5 Pa. The cross-sectional area rate of change in the distal region has a much different curve than the proximal region. For segment C, there is an increasing trend which continues to increase in rate over the weeks, whereas for segment D, there is an increasing trend which decreases in rate over the weeks. The venous swing segment (segment A) has the highest temporal WSS fluctuations and the highest magnitude of all segments, averaging approximately 9 Pa with a range of 6 Pa. The cross-sectional area increases slightly over the weeks.

Little remodelling is seen in segment E, due to retrograde flow in the outflow artery remaining almost constant week to week. The TAWSS in this segment has a temporally average value of 2 Pa and has the smallest fluctuations of all segments. The area also has the smallest fluctuations, but appears to increase (marginally), as the weeks progress.

### Conclusion

The geometric and haemodynamic timeline was established for a successful AVF maturation, with data taken weekly for a duration of 15 weeks. The use of a large temporal data set highlighted the variance found in AVF remodelling, particularly in the week-to-week measurement of the blood flow rates, and the potential for misleading results from only using a limited number of time points when scanning an AVF during maturation.

It was found that the largest changes occurred within the first two weeks of the creation, but it was noted that there were still outward remodelling and flow changes in the later weeks. A key finding was that the vein and the artery remodel at separate rates to each other, but also at different rates based on proximity to the anastomosis. The inflow artery in the proximal location and distal location had much different rates, where the proximal region seemed to converge towards a value and the distal location was increasing in trend.

This study provides further evidence that wall shear stress at time of creation could be a useful predictor for AVF success or failure. The WSS values for this patient did not change significantly from the measured value in week 1. In addition, the TAWSS did not restore to baseline (pre-creation) values and remained elevated at a new set-point level, which suggests that the set-point level for TAWSS is able to adapt after an AVF creation. The constancy of WSS was more evident in the proximal regions. However, there were higher TAWSS fluctuations throughout the cardiac cycle and temporally (week to week) in regions near (less than 40 mm away) to the anastomosis for both the vein and artery indicating instability in this region.

Disturbance of flow was confined to regions near to the anastomosis and in the venous swing segment. It was found that in this patient continued exposure to flow disturbances, in the form of WSS metrics, was not detrimental to the success of the maturation.

## Footnotes

1 Magnetic resonance imaging.
